# Neuromeningeal Cryptococcosis and Tuberculosis Coinfection in Bamako: A One-Year Case Series

**DOI:** 10.1093/ofid/ofad438

**Published:** 2023-08-18

**Authors:** Dramane Ouedraogo, Yacouba Cissoko, Mariam Soumare, Amavi Essénam Alle Akakpo, Ouo-Ouo Loua, Issa Konate, Safiatou Niare Doumbo, Sounkalo Dao

**Affiliations:** Department of Infectious Diseases and Tropical Medicine, Point “G” University Teaching Hospital, Bamako, Mali; Department of Infectious Diseases and Tropical Medicine, Point “G” University Teaching Hospital, Bamako, Mali; University Clinical Research Center (UCRC) Laboratory, University of Sciences, Techniques and Technologies of Bamako (USTTB), Bamako, Mali; Department of Infectious Diseases and Tropical Medicine, Point “G” University Teaching Hospital, Bamako, Mali; Department of Infectious Diseases and Tropical Medicine, Point “G” University Teaching Hospital, Bamako, Mali; Department of Infectious Diseases and Tropical Medicine, Point “G” University Teaching Hospital, Bamako, Mali; Department of Infectious Diseases and Tropical Medicine, Point “G” University Teaching Hospital, Bamako, Mali; University Clinical Research Center (UCRC) Laboratory, University of Sciences, Techniques and Technologies of Bamako (USTTB), Bamako, Mali; Department of Parasitic Diseases Epidemiology, Faculty of Medicine and Dentistry, University of Sciences, Techniques and Technologies of Bamako (USTTB), Bamako, Mali; Department of Infectious Diseases and Tropical Medicine, Point “G” University Teaching Hospital, Bamako, Mali; University Clinical Research Center (UCRC) Laboratory, University of Sciences, Techniques and Technologies of Bamako (USTTB), Bamako, Mali

**Keywords:** coinfection, cryptococcosis, HIV, tuberculosis

## Abstract

Cryptococcosis and tuberculosis are life-threatening opportunistic infections that occur in apparently immunocompetent or severely immunocompromised individuals worldwide. As both infections are strongly linked to HIV infection, they may share certain clinical manifestations, and the interaction of their treatments should be considered. However, despite their similarity, concurrent tuberculosis and cryptococcal infections have rarely been reported in West Africa. Herein, we present 3 cases of neuromeningeal cryptococcosis and lung tuberculosis coinfection collected prospectively over a year at the Department of Infectious Diseases of the Point G Teaching Hospital in Bamako. Two patients had HIV disease, and the third patient had no underlying immunosuppressive illnesses. Thus, active screening for tuberculosis and cryptococcosis, particularly in individuals with HIV, can reduce misdiagnosis and ensure appropriate coinfection management. Moreover, this may reduce mortality due to AIDS-related opportunistic infections in resource-limited settings.

Cryptococcosis is an invasive fungal infection caused by the yeast *Cryptococcus* that occurs globally [1]. It is an opportunistic infection mostly affecting patients with impaired cell-mediated immunity [1]. In people with HIV (PWH), cryptococcal meningitis (CM) is an AIDS-defining illness, usually occurring when the CD4+ cell count is <100 cells/mL. In 2014, the global estimated incident cases of cryptococcosis were 223 100, with Sub-Saharan Africa accounting for 162 500 cases (73%) [2].

Tuberculosis (TB) is caused by the bacillus *Mycobacterium tuberculosis* and is a communicable disease that is a leading cause of illness and death worldwide [3]. It is the most common opportunistic infection in PWH and is likely the leading cause of death in Sub-Saharan Africa [4].

Cryptococcosis and TB are life-threatening infections that affect people worldwide. They are strongly linked to HIV infection and may share some clinical features [5]. According to a systematic review, several cases of cryptococcal/TB coinfection have been reported worldwide over long periods, including 193 cases over 60 years in China [6], 23 cases over 14 years in Taiwan [5], 5 cases over 12 years in India [7], and 184 cases over 7 years in South Africa and Uganda [8]. However, in West Africa, cryptococcal/TB coinfection is underreported and poorly described. Three cases were reported over an 8-year period in Ivory Coast [9], and 1 case was reported in 2020 in Senegal [10]. Herein, we present a series of 3 cases of cryptococcal/TB coinfection who were prospectively recruited over a 1-year period at the Department of Infectious and Tropical Diseases of the Point G Teaching Hospital in Bamako (Mali).

## CASES PRESENTATION

From January to December 2022, we diagnosed 3 cases of cryptococcal/TB coinfection (Table 1). The median age of the patients (range) was 27.3 (18–32) years, with 2 of the cases being women. Two of the 3 patients were PWH, with a median CD4+ cell count of 74 cells/µL and a median viral load of 46 560 copies/mL. One of the PWH (Case 3) was not undergoing antiretroviral therapy (ART) at the time of admission and started ART 30 days after starting treatment for opportunistic infections. The patient without HIV (Case 1) did not have any other immunocompromizing conditions aside from TB. One of the patients with HIV (Case 2) had a previous history of cured TB infection, while the other 2 patients showed no TB history. Clinically, all the patients exhibited chronic cough and fever and weight loss, with 1 patient reporting neck stiffness and seizures. All the patients were treated in accordance with the World Health Organization (WHO) guidelines for TB. However, for cryptococcosis, fluconazole monotherapy (400-mg infusion 3 times daily for 14 days followed by 400 mg orally once daily) was administered because the WHO-recommended amphotericin B and flucytosine were unavailable in Mali. The patient without HIV infection died after a 9-week follow-up, and the patients with HIV infection survived.

**Table 1.** Epidemiological and Clinical Characteristics of Four Cases of Cryptococcosis/Tuberculosis Coinfection in Bamako, Mali

…	Case 1	Case 2	Case 3
Age	18 y	32 y	32 y
Sex	F	M	F
Underlying diseases	No	HIV	HIV
ART; days on ART at admission	N/A	Yes; 37 d	No
CD4	750 cells/μL	9 cells/μL	138 cells/μL
HIV viral load	N/A	93 079 copies/mL	40 copies/mL
Pigeon contact	No	No	No
TB diagnostics	Xpert MTB/RIF (+) on gastric aspirate fluid	Xpert MTB/RIF (+) on sputum	Xpert MTB/RIF (+) on sputum and CSF
CM diagnostics, species	CSF: CrAg (+), India ink microscopy (+), culture: *C. neoformans*	CSF: CrAg (+), India ink microscopy (+), culture: *C. neoformans*	CSF: CrAg (+), India ink microscopy (+), culture: *C. neoformans*
Time between the 2 diagnoses	3 d	64 d	1 d
Anti-TB treatment	2 mo of HRZE and 4 mo of RH	2 mo of HRZE and 4 mo of RH	2 mo of HRZE and 10 mo of RH
Antifungal treatment	FCZ: induction therapy (1200 mg/d for 14 d) and maintenance therapy (800 mg/d for 8 wk)	FCZ: induction therapy (1200 mg/d for 14 d) and maintenance therapy (800 mg/d for 8 wk)	FCZ: induction therapy (1200 mg/d for 14 d) and maintenance therapy (800 mg/d for 8 wk)
Outcome	Died	Alive	Alive

Abbreviations: ART, antiretroviral therapy; CM, cryptococcal meningitis; CrAg, cryptococcal antigen; CSF, cerebrospinal fluid; E, ethambutol; FCZ, fluconazole; H, isoniazid; N/A, not applicable; R, rifampicin; TB, tuberculosis; Z, pyrazinamide.

### Case 1

An 18-year-old woman presented with chronic fever and cough and meningitis symptoms, including neck stiffness and a Glasgow Coma Scale score of 8/15. Her CD4+ cell count was 750 cells/mL. The patient showed progressive weight loss, presenting with a body mass index (BMI) of 13.6 kg/m^2^ at the time of admission. TB was initially diagnosed by a positive Xpert *M. tuberculosis*/resistance to rifampin (MTB/RIF) test via gastric aspiration. After 3 days, microscopy of a cerebrospinal fluid (CSF) sample stained with India ink revealed positive results. In addition, CSF examination showed a total cell count of 10 cells/mm^3^ with 60% polymorphs and 40% lymphocytes. The CSF glucose and protein levels were 2.34 mmol/L and 0.89 g/L, respectively. Additionally, cryptococcal antigen (CrAg) testing of the CSF sample yielded positive results. A fungal culture of the CSF sample using Sabouraud's medium revealed the presence of *Cryptococcus neoformans*. Blood tests revealed normal blood glucose levels (2 fasting blood glucose levels at 0.97 and 0.92 g/L) and hypoproteinemia at 47.61 g/L. The patient did not have any underlying immunosuppressive diseases and demonstrated negative HIV serology when repeated 3 times, normal hemoglobin electrophoresis results (hemoglobin A [Hb-A], 97.8%; Hb-A2, 2.2%), and normal cancer marker levels (alpha-fetoprotein, 6.5 ng/mL; cancer antigen 125, 27 U/mL; cancer antigen 15–3, 18 U/mL; carbohydrate antigen 19–9, 12 U/mL; and carcinoembryonic antigen, 0.92 ng/mL). The patient was started on TB treatment and fluconazole. She died 9 weeks after admission.

### Case 2

A 32-year-old man with HIV was undergoing ART (including tenofovir disoproxil 300 mg, lamivudine 300 mg, and dolutegravir 50 mg) for 37 days before admission. He showed a previous history of bacteriologically confirmed pulmonary TB, which was successfully treated, and was declared to be cured in 2021. A CM diagnosis was suspected owing to the presence of persistent headaches and was confirmed via positive results of direct microscopy with India ink staining and CSF culture. Additionally, a CSF sample was positive for CrAg. Furthermore, CSF examination revealed a total cell count of 1 cell/mm^3^, CSF glucose level of 1.83 mmol/L, and protein level of 0.5 g/L. The patient’s CD4+ cell count was 9 cells/mL, and his viral load was 93 079 copies/mL. While receiving anticryptococcal treatment at the consolidation stage, the patient experienced chronic cough, intermittent and night fever, and weight loss (BMI, 14.45 kg/m^2^). Sputum samples tested positive for the acid-fast bacilli and Xpert MTB/RIF tests, with subsequent culture confirming the presence of *M. tuberculosis*, leading to diagnosis of recurrent TB. In addition to cryptococcosis treatment, the patient was started on a TB treatment regimen, which involved 2 months of HRZE (isoniazid, rifampicin, pyrazinamide, and ethambutol) followed by 4 months of HR (isoniazid and rifampin). The patient continued receiving ART with an additional dose of dolutegravir (50 mg/d) owing to the interaction of dolutegravir and rifampicin. The clinical outcome of the patient was favorable, and he was discharged from the hospital. At the fifth month of follow-up after discharge, the BMI of the patient increased to 18.8 kg/m^2^, indicating good prognosis.

### Case 3

A 32-year-old woman who was newly diagnosed with HIV presented with a persistent cough, prolonged evening fever, fatigue, unexplained weight loss (>10% of body weight), and night sweats. The BMI, CD4+ cell count, and viral load of the patient were 21.30 kg/m^2^, 138 cells/mL, and 40 copies/mL, respectively. Chest x-ray revealed miliary TB. Sputum and CSF samples were positive using the Xpert MTB/RIF test. Furthermore, a CSF sample was positive for CrAg. Direct microscopy with India ink staining of the CSF sample showed positive results, with subsequent culture revealing the presence of *C. neoformans.* The white blood cell count and protein concentration in the CSF sample were 400 cells/μL (10% lymphocytes) and 0.90 mg/dL, respectively. Accordingly, the patient was diagnosed with concurrent pulmonary TB, tuberculous meningitis, and CM and started on TB treatment for 12 months and cryptococcosis treatment, showing good compliance. A month later, ART with tenofovir disoproxil 300 mg, lamivudine 300 mg, and dolutegravir 100 mg was initiated. The patient was alive at the second month of follow-up, demonstrating good clinical improvement and weight gain (BMI, 22.77 kg/m^2^).

## DISCUSSION

Cryptococcosis and TB are opportunistic infections often occurring in patients with severe immunodeficiency [1, 5, 7]. Both diseases share endemic characteristics, as well as certain clinical manifestations, in Sub-Saharan Africa and Asian countries [5, 11].

Only a few cases of cryptococcal/TB coinfection have been reported in West Africa [10]. This 1-year study revealed that cryptococcal/TB coinfection is not rare in Mali, particularly in PWH. Two of the 3 patients in this study were PWH. Cryptococcosis usually occurs in PWH with CD4+ cell counts of <100 and TB at any stage of immunodeficiency [1, 12]. Herein, the patients with HIV exhibited advanced immunosuppression, with a median CD4+ cell count of <100 cells/mm^3^ at the time of diagnosis. HIV infection is the most common comorbidity in patients who develop these opportunistic infections [5, 7, 11, 13]. However, in 1 patient in this study, no underlying disease or immunosuppression status was identified upon investigation. Other such cases have been reported in the literature [5, 7], including rare immunodeficiency disorders such as idiopathic CD4+ lymphocytopenia, which may predispose patients to cryptococcal/TB coinfection [5, 14]. In addition, previous studies have reported that TB infection impairs cellular immunity and is recognized as a predisposing factor for developing cryptococcosis [5, 15, 16]. Herein, in a PWH (Case 2), CM/TB coinfection occurred 37 days after the start of ART. Ellis et al. reported similar findings in their case series involving patients with CM/TB meningitis coinfection who were PWH in Uganda [11]. Cryptococcosis or TB, particularly of the central nervous system, that occurs shortly after starting ART is a clinical argument for an unmasked immune reconstitution inflammatory syndrome (IRIS) [11]. IRIS is a potential complication of ART initiation. It constitutes a state of hyperinflammation against latent infections that occurs following improvement in CD4+ cell count and immune response at the start of HIV therapy [17]. Mycobacteria and cryptococci are 2 of the most frequently reported opportunistic pathogens. Therefore, it is essential to determine whether patients with HIV/AIDS experience latent infections before starting ART [17]. Current guidelines recommend starting ART within 2 weeks of diagnosing most opportunistic infections. However, special consideration should be given to opportunistic infections of the central nervous system (eg, cryptococcosis and tuberculous meningitis) [17–19]. The introduction of the Xpert MTB/RIF test has considerably improved TB diagnosis. Cryptococcosis is usually diagnosed via microscopy, serology, or culture. However, India ink stain microscopy is an inexpensive and rapid technique for identifying *Cryptococcus* species in CSF and other bodily fluids [1]. CrAg detection in the blood is important for CM diagnosis and is currently recommended by the WHO for screening PWH with CD4+ cell counts of ≤100 cells/mL before starting or resuming ART [20]. However, this WHO-recommended systematic screening test is ineffective in our country owing to its low availability. Thus, only patients with relevant symptoms are assessed for cryptococcal infection.

Cryptococcosis and TB are AIDS-defining illnesses [5]. Therefore, treatment of these infections and ART are strongly recommended. However, treatment is prolonged for both infections, leading to high costs and adverse effects, as well as poor compliance and increased antimicrobial resistance [7]. The WHO guidelines for 2022 recommend antifungal combinations (amphotericin B, flucytosine, and fluconazole) for 2 weeks as initial induction therapy for CM [19]. However, the availability of amphotericin B and flucytosine in Africa is limited [21], particularly in Mali. Thus, we used high-dose fluconazole monotherapy as the induction therapy. Although previous studies of CM have reported that high-dose fluconazole monotherapy reduces survival [21–24], this was the only option in our setting. In addition, 2 of our 3 patients responded favorably to this treatment. According to the WHO guidelines, new pulmonary TB patients should receive a regimen containing 6 months of rifampicin (2HRZE/4HR), and those with tuberculous meningitis should receive a regimen containing 12 months of rifampicin (2HRZE/10HR) [25, 26]. In the treatment of TB/CM coinfection, it is important to consider the interaction between rifampicin and fluconazole, whose concurrent use reduces the half-life and the area under the plasma concentration–time curve of fluconazole [27–29]. Therefore, when rifampicin is administered alongside fluconazole, a 30% increase in fluconazole dose should be considered, particularly during the 200-mg/d treatment period, for treating serious infections [27]. All patients with HIV responded favorably to the concomitant antifungal and antituberculosis therapy. Delays in diagnosis and treatment can be fatal for patients with cryptococcosis or TB, particularly PWH [5]. The coexistence of other opportunistic diseases alongside CM is a negative prognostic factor for PWH [9]. High mortality rates have been reported in several case series involving patients with cryptococcal/TB coinfection, such as in India (2 deaths out of 5 cases) [7], China (2 deaths out of 8 cases) [30], Uganda (3 deaths out of 5 cases) [11], and Senegal (1 death out of 1 case) [10]. However, herein, the outcome for PWH was favorable, suggesting that early diagnosis and treatment of cryptococcal/TB coinfection can benefit PWH.

Thus, cryptococcosis/TB coinfection appears to be generally uncommon. However, this 1-year case series has revealed that this coinfection is not rare in Mali. Therefore, it is necessary to actively screen for TB and cryptococcosis, particularly in PWH, to reduce infection-related mortality in resource-limited settings.
